# Patient-reported barriers to osteoporosis therapy

**DOI:** 10.1007/s11657-016-0272-5

**Published:** 2016-04-29

**Authors:** Brianna R. Lindsay, Temitope Olufade, Jennifer Bauer, Jane Babrowicz, Rebecca Hahn

**Affiliations:** 1 Center for Observational and Real-World Evidence, Merck & Co. Inc., Kenilworth, NJ USA; 2 Nielsen, New York, NY USA

**Keywords:** Osteoporosis, postmenopausal/drug therapy, Medication adherence, Patient compliance, Persistence, Health knowledge, attitudes, practice, Health surveys

## Abstract

**Summary:**

We investigated reasons for non-treatment of osteoporosis and discontinuation of osteoporosis therapy. Barriers to treatment include patients’ preference for alternative treatments and a fear of possible side effects. Side effects are a common reason for treatment discontinuation, and they may be associated with a lack of willingness to restart treatment.

**Purpose/introduction:**

Osteoporosis patients commonly cite treatment-related side effects, or the fear thereof, as a reason for discontinuing or not initiating anti-osteoporosis medications. The purpose of this study was to investigate, from the patient’s perspective, reasons for (i) non-treatment of osteoporosis and (ii) discontinuation of osteoporosis therapy.

**Methods:**

This was an internet-based survey of postmenopausal women in the USA who self-reported having been diagnosed with osteoporosis. Respondents were recruited from consumer research panels and received nominal compensation.

**Results:**

Within the surveyed population (*N* = 1407), 581 patients were currently being treated, 503 had never been treated, and 323 had previously been treated. Among patients never treated for osteoporosis, the highest ranking reasons for non-treatment were the use of alternative treatments such as over-the-counter vitamins/supplements (57.5 % of respondents) and fear of side effects (43.9 %). Among previously treated patients, frequent reasons for discontinuation included the direction of the physician (41.2 % of respondents), concerns about long-term safety (30.3 %), and the experience of side effects (29.8 %). When asked about their willingness to restart their osteoporosis medication, previously treated patients who were not willing (*N* = 104) to restart had a higher frequency of experiencing side effects (44.2 versus 20.5 % of those willing; *P* < 0.001).

**Conclusions:**

From the osteoporosis patient’s perspective, barriers to prescription treatment include a preference for alternative, non-prescription treatments and a fear of possible side effects. Side effects are one of the most common reasons for discontinuing osteoporosis medications, and they appear to be associated with a lack of willingness to restart treatment.

**Electronic supplementary material:**

The online version of this article (doi:10.1007/s11657-016-0272-5) contains supplementary material, which is available to authorized users.

## Introduction

The estimated prevalence of osteoporosis among US women aged 50 and older, based on bone mineral density of the total hip or of the total hip or spine, was 14–16 % in 2010 [1]. The National Osteoporosis Foundation recommends pharmacologic treatment in patients with low bone mineral density, corresponding to *T* scores ≤−2.5, at the femoral neck, total hip, or lumbar spine [2]. However, despite this recommendation, substantial under-treatment of osteoporosis in postmenopausal US women has been observed [3, 4]. A 2014 retrospective cohort study of US women aged ≥55 with a claims-documented diagnosis of osteoporosis found that 64.3 % of patients did not receive osteoporosis treatment within 1 year after the diagnosis [3].

For those patients initiating therapy, discontinuation is commonly reported. A retrospective cohort study on US women aged ≥55 years initiating osteoporosis therapy reported discontinuation rates of 45, 58, and 70 % at 6, 12, and 24 months, respectively [5]. Similar 12-month discontinuation rates were reported in other US studies [6, 7]. Many discontinuations occur because of side effects [8], but some may occur in the context of a drug holiday, that is, an intentional discontinuation of medication recommended by national guidelines for patients with satisfactory response to long-term therapy [9]. Whether patients reinitiate therapy after discontinuation has been addressed by several US studies, with rates of reinitiation ranging from 30 % within 6 months to 50 % within 2 years [5, 6, 10, 11].

Previous studies have identified several patient characteristics, e.g., body mass index [12] and comorbidities [13], that are associated with non-treatment. Similarly, correlates of reinitiation of therapy have been identified, including longer duration of treatment before the discontinuation, younger age, and a history of fracture [5, 10]. This study was designed to complement these findings by assessing the reasons for non-treatment and the reasons for discontinuation from the patient’s perspective, with an emphasis on how the reasons for discontinuation interact with a patient’s willingness to reinitiate therapy.

## Methods

### Study design and data source

This was a cross-sectional online survey of women in the USA who participated in online consumer panels, primarily Harris Poll Online. This consumer panel consists of adults, aged 18 or older, who have self-selected into the panel because they are interested in participating in online surveys. The survey was conducted between July 7 and July 25, 2014. Eligible participants received an invitation via e-mail with information about accessing the online survey. Potential participants were offered compensation in the form of panel points, which could be redeemed for a nominal monetary reward.

### Survey participants

Postmenopausal US women aged ≥40 who self-reported being diagnosed with osteoporosis were eligible for inclusion. Participants were classified by self-report as currently treated, never treated, and previously treated. Possible treatments included both oral and injectable prescription medications.

### Survey content

The survey was pre-tested among a small group of about 12 adults in order to test the survey with live respondents and allow the added benefit of qualitative insight into patient perceptions and attitudes. This ensured that the survey was clear and concise while capturing the most relevant dimensions necessary for comprehensive analysis (see Appendix for survey).

Respondents were asked about their current and past osteoporosis treatments, their reasons for not starting or for discontinuing treatment, their interactions with their treating physicians, and, if previously treated, their willingness to restart therapy. Demographic information was also collected, including comorbidities, history of fractures, race/ethnicity, education, and health insurance coverage.

Never-treated patients were asked to select all their reasons for remaining untreated and to rank their top three reasons. Possible choices included employing alternative treatments or implementing lifestyle changes, various beliefs about osteoporosis or medication, concerns about safety, and cost issues. Previously treated patients were asked to select all their reasons for discontinuing treatment and to rank their top three reasons. Possible choices included concerns about side effects, cost and insurance coverage, and dosing or regimen. Previously treated patients were also queried about their willingness to restart the medication originally used to treat osteoporosis. Responses included not at all willing, somewhat willing, willing, very wiling, and extremely willing. All patients were asked to assess how often statements about their interactions with their treating physician were true. Statements focused on the relationship and trust between the patient and their physician. Responses included never, rarely, sometimes, often, and always.

### Data analysis

A total sample size of 1400 was predicted to be sufficient to detect statistically significant differences between the patient subgroups. In order to ensure an adequate sample size for analyses, quotas were set for the three categories of respondents based on prevalence estimates from previous studies: *N* = 500 for never treated (∼35 %), *N* = 320 for previously treated (∼23 %), and *N* = 580 for currently treated patients (∼42 %). Participants were continuously recruited until quotas were met. The analyses were primarily descriptive, with bivariate statistical tests used as needed. Descriptive data for each of the three groups of patients are presented as numbers and percentages for categorical variables or as means and standard deviations for continuous variables. Patient characteristics were compared across all three treatment groups. Perceptions of the physician-patient relationship among currently treated patients were compared with those of never treated and previously treated patients. Reasons for discontinuation were compared between patients not willing to restart therapy and somewhat to extremely willing to restart therapy. Throughout the analyses, continuous variables were compared with *t* tests and categorical variables with chi-square tests or analysis of variance (ANOVA) techniques, and *P* values <0.05 were considered statistically significant.

## Results

### Characteristics of the study population

A total of 1407 osteoporosis patients participated in the survey (Table 1). The mean age of all patients was 64 years. Most patients had at least some college or graduate schooling (71.3 % total), had drug coverage through their health insurance (89.0 %), and reported having had a DEXA scan (83.5 %). The majority of patients included in this study were white (93.2 %; data not shown). Furthermore, patients were almost equally distributed between regions of the USA with 36.2 % in the south, 23.5 % in the midwest, 20.3 % in the northeast, and 20.0 % in the west (data not shown).

**Table 1:** Baseline characteristics of survey respondents

	Total *N* = 1407	Never treated *N* = 503	Previously treated *N* = 323	Currently treated *N* = 581	*P* value
Age, mean (SD) years	64 (9.1)	62 (8.7)	68 (8.3)	63 (9.2)	<0.001
Years since diagnosis, mean (SD)	7.8 (6.7)	6.5 (6.1)	10.8 (7.5)	7.2 (6.3)	<0.001
Education					
High school graduate or less	395 (28.1)	163 (32.4)	85 (26.3)	147 (25.3)	0.004
Some college or college graduate	730 (51.9)	262 (52.2)	169 (52.3)	299 (51.5)	
Graduate school	273 (19.4)	73 (14.5)	68 (21.1)	132 (22.7)	
Drug coverage					
Yes, through my health insurance	1252 (89.0)	422 (83.9)	286 (88.5)	544 (93.6)	<0.001
Yes, through a patient assistance or co-pay card	49 (3.5)	19 (3.8)	14 (4.3)	16 (2.8)	
No	95 (6.8)	56 (11.1)	22 (6.8)	17 (2.9)	
Not sure	11 (0.8)	6 (1.2)	1 (0.3)	4 (0.7)	
General health					0.004
Poor/fair	464 (33.0)	192 (38.2)	93 (28.8)	179 (30.8)	
Good	486 (34.5)	176 (35.0)	119 (36.8)	191 (32.9)	
Very good/excellent	457 (32.5)	135 (26.8)	111 (34.4)	211 (36.3)	
Number of poor physical health days during past 30 days, mean (SD)	7.27 (10.01)	8.09 (10.22)	6.42 (9.73)	7.03 (9.96)	0.095
Number of poor mental health days during past 30 days, mean (SD)	5.14 (8.57)	6.14 (9.31)	5.00 (8.58)	4.35 (7.79)	0.001
Number of days poor physical or mental health prevented usual activities during past 30 days, mean (SD)	5.32 (9.04)	6.46 (9.75)	4.58 (8.57)	4.75 (8.55)	0.002
DEXA scan	1175 (83.5)	372 (74.0)	285 (88.2)	518 (89.2)	<0.001
Broke a bone after age 45	539 (38.3)	177 (35.2)	130 (40.2)	232 (39.9)	0.183
Fell in the past 12 months	466 (33.1)	177 (35.2)	106 (32.8)	183 (31.5)	0.433

Values are presented as *N* (%) unless indicated otherwise

*DEXA* dual X-ray absorptiometry, *SD* standard deviation

Within the surveyed population, 581 patients were currently being treated, 503 had never been treated, and 323 had previously been treated (Table 1). Currently treated patients more frequently had graduate level education (22.7 versus 14.5 % for never-treated and 21.1 % for previously treated patients; *P* = 0.004), had drug coverage through insurance (93.6 versus 83.9 % for never-treated and 88.5 % for previously treated patients; *P* < 0.001), and reported having had a DEXA scan (89.2 versus 74.0 % for never-treated and 88.2 % for previously treated patients; *P* < 0.001).

### Reasons for non-treatment

**Fig. 1:**
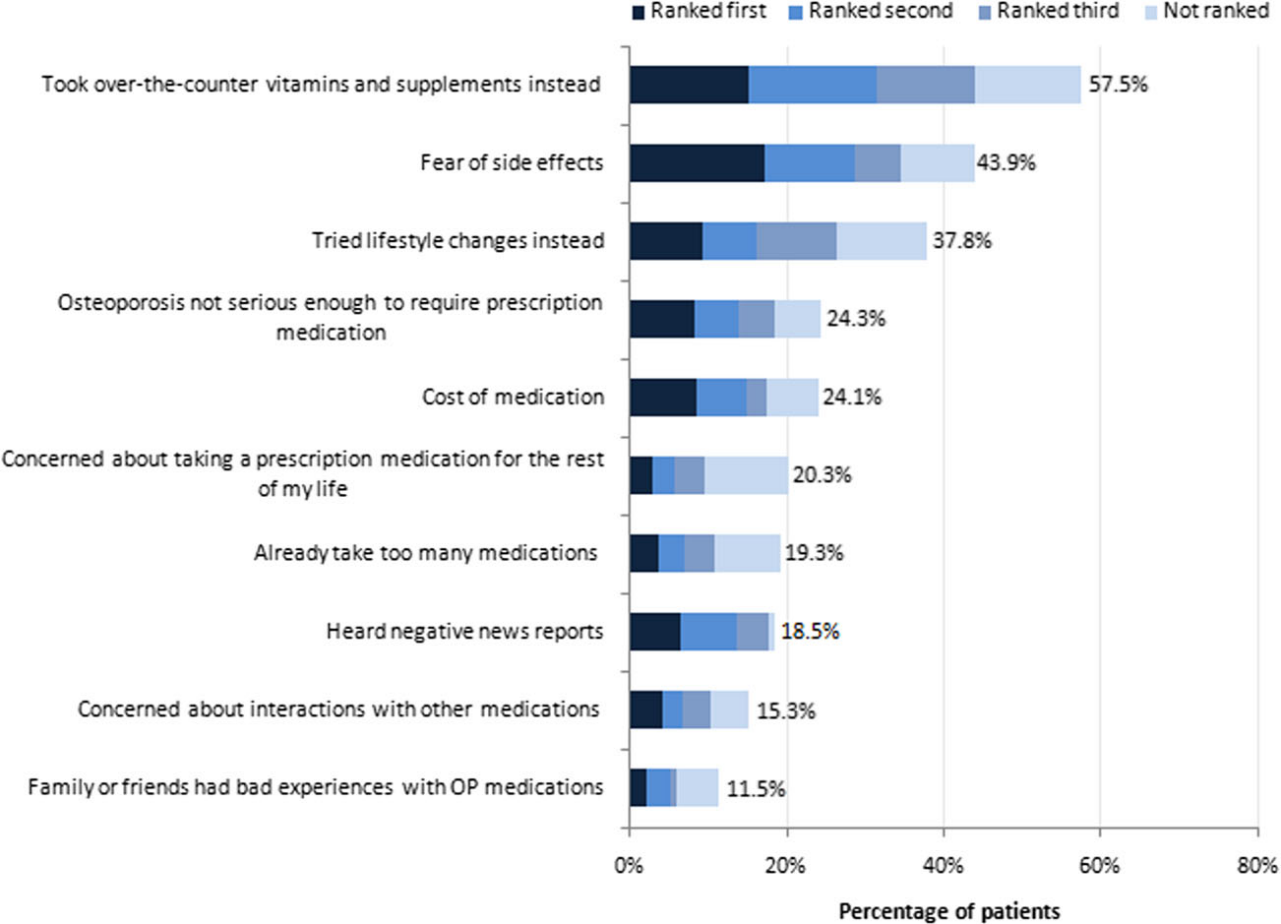
Reasons for not initiating osteoporosis treatment among untreated patients. *OP* osteoporosis. *N* = 503

**Fig. 2:**
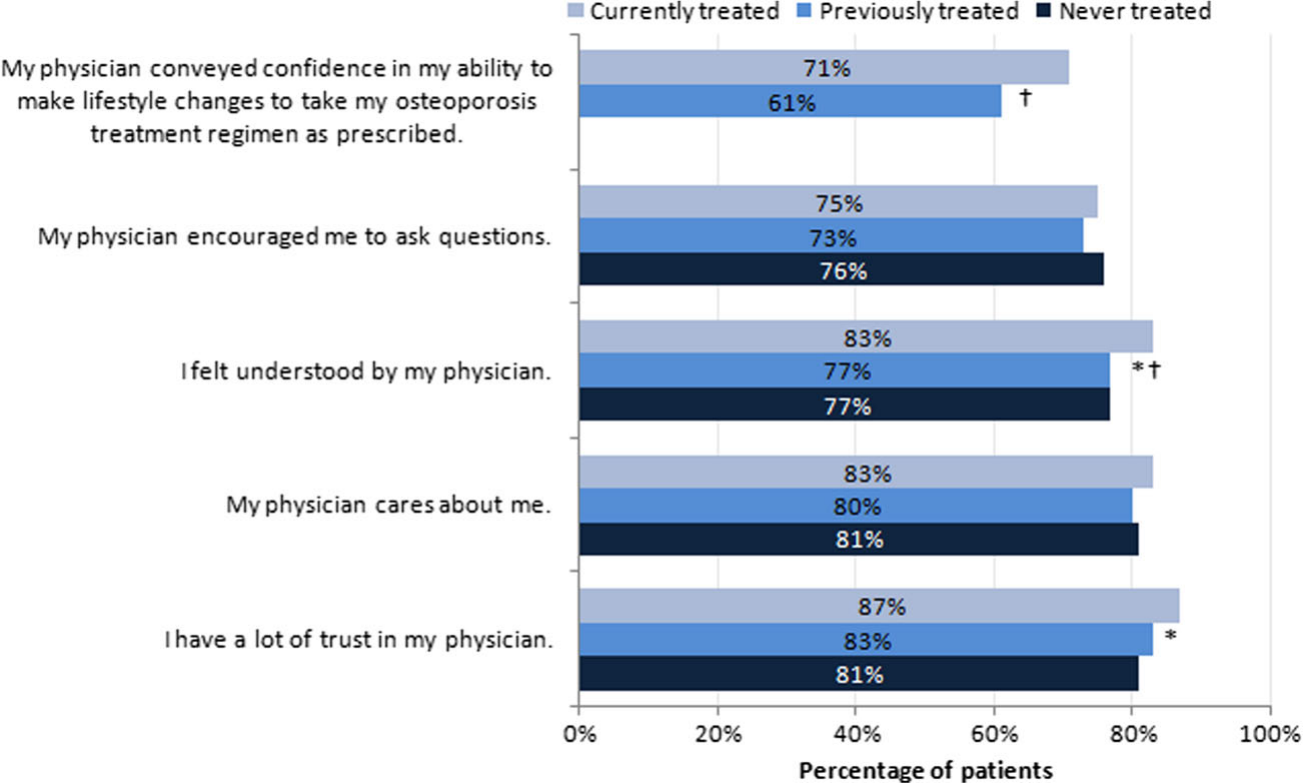
Perceptions of the physician-patient relationship. *OP* osteoporosis. The *bars* show the percentage of women who responded either “often” or “always” when asked to indicate how often each statement was true regarding visits with the physician who treated their osteoporosis over the past 12 months. *P < 0.05 between currently treated and never-treated patients. ^†^ *P* < 0.05 between currently treated and previously treated patients

Among patients never treated for osteoporosis, the highest-ranking reasons for non-treatment were use of over-the-counter vitamins/supplements instead (57.5 % of respondents), fear of side effects (43.9 % of respondents), and implementation of lifestyle changes instead (37.8 % of respondents; Fig. 1). Other common reasons included the belief that osteoporosis is not serious enough to require prescription medication (24.3 % of respondents) and concerns about the cost of medication (24.1 % of respondents). Never-treated patients were less likely than currently treated patients to feel understood by their physicians (77 versus 83 %; *P* < 0.05) and to have “a lot of trust” in their physicians (81 versus 87 %; *P* < 0.05; Fig. 2).

### Reasons for discontinuation

**Fig. 3:**
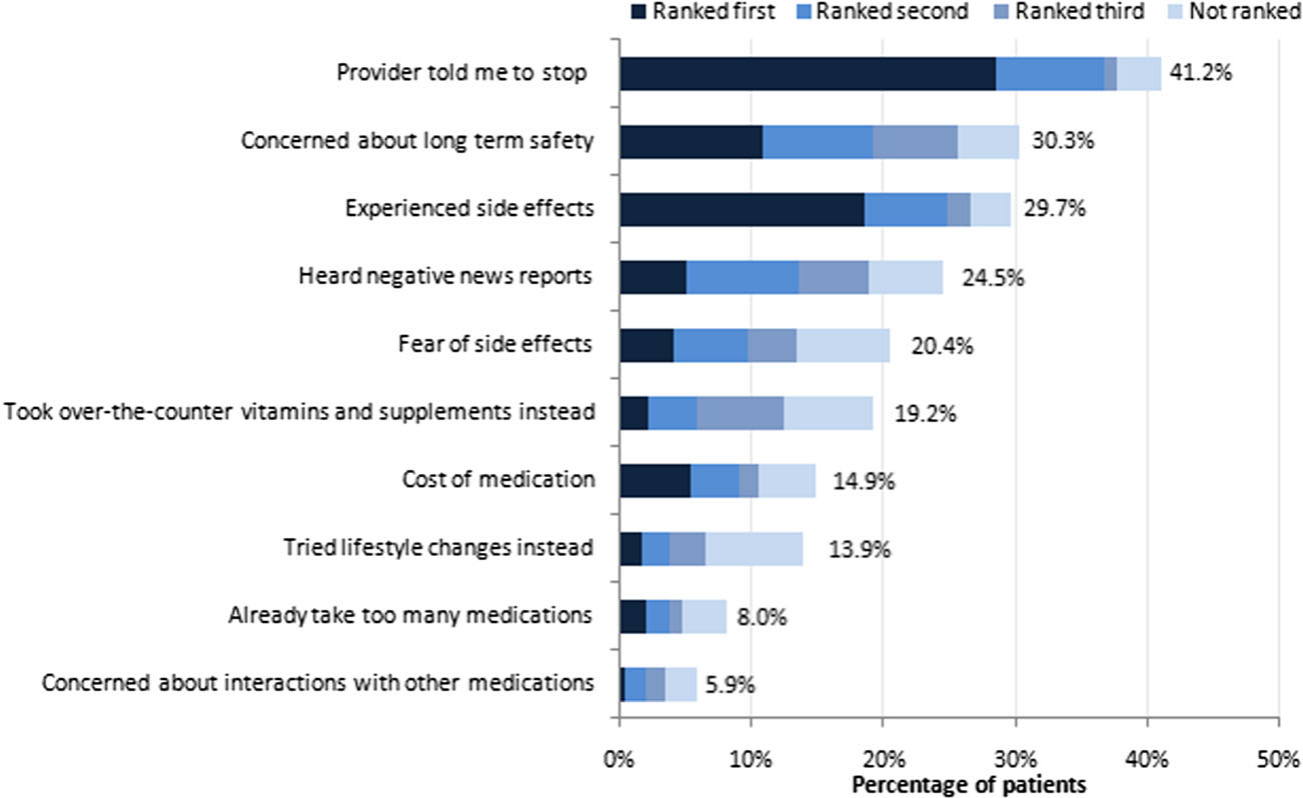
Reasons for discontinuing osteoporosis therapy among previously treated patients. *N* = 323

Among previously treated patients, the most frequent reason for discontinuation was the direction of the physician (41.2 % of respondents; Fig. 3). Concerns about long-term safety (30.3 %) and the experience of side effects (29.7 %) also ranked high among the reasons for discontinuation. Previously treated patients were less likely than currently treated patients to feel understood by their physicians (77 versus 83 %; *P* < 0.05) and less likely to say that their physician expressed confidence in their ability to take the medication as prescribed (61 versus 71 %; *P* < 0.05; Fig. 2).

### Willingness to reinitiate treatment

Patients not at all willing (*N* = 104) differed from those somewhat to extremely willing (*N* = 195) to restart their medication in terms of their reasons for discontinuation (Table 2). Among these reasons, patients who were not willing to restart had a higher frequency of experiencing side effects (44.2 versus 20.5 % of those willing; *P* < 0.001), had more often heard negative news about osteoporosis medications (33.7 versus 20.5 % of those willing; *P* = 0.013), and more frequently had concerns about the long-term safety of their osteoporosis medications (40.4 versus 25.6 % of those willing; *P* = 0.009). Patients somewhat to extremely willing to restart therapy were more likely to have discontinued for reasons of cost than those not willing to restart (18.5 versus 8.7 %; *P* = 0.024).

**Table 2:** Reasons for discontinuation among patients willing or not willing to restart therapy

Reason for discontinuation	Not willing *N* = 104	Somewhat to extremely willing *N* = 195	*P* value
Perceived side effects			
I experienced side effects	*46 (44.2)*	*40 (20.5)*	*<0.001*
I heard negative news reports about OP medications	*35 (33.7)*	*40 (20.5)*	*0.013*
Fear of side effects	25 (24.0)	35 (17.9)	0.211
I knew family or friends who had bad experiences with OP medications	*10 (9.6)*	*5 (2.6)*	*0.008*
Safety			
I was concerned about long-term safety	*42 (40.4)*	*50 (25.6)*	*0.009*
I was concerned about interactions with other medications	4 (3.8)	14 (7.2)	0.248
Cost/coverage			
Cost of medication	*9 (8.7)*	*36 (18.5)*	*0.024*
It wasn’t covered by my insurance	2 (1.9)	14 (7.2)	0.054
Dosing/regimen			
My health care provider told me to stop taking the OP prescription	43 (41.3)	80 (41.0)	0.957
I felt like I already take too many medications	7 (6.7)	16 (8.2)	0.649
Inconvenient/complex dosing	1 (1.0)	2 (1.0)	–
Problems remembering to take it	1 (1.0)	11 (5.6)	0.050
Beliefs about osteoporosis/medication			
I did not think the prescription would work/was effective	6 (5.8)	11 (5.6)	0.9636
I did not believe that my osteoporosis was serious enough to take a medication	3 (2.9)	12 (6.2)	0.2174
I did not understand the purpose of the medication	0 (0.0)	3 (1.5)	–
I did not believe that my OP was life threatening	4 (3.8)	12 (6.2)	0.3984
I did not think that I needed OP medication	4 (3.8)	9 (4.6)	0.7561
Alternatives			
I decided to take over-the-counter vitamins and supplements for my osteoporosis instead of prescription medication	22 (21.2)	32 (16.4)	0.3098
I decided to try lifestyle changes instead of taking OP medications	20 (19.2)	22 (11.3)	0.0596

Values are presented as *N* (%). Italicized data indicate statistically significant differences between patients not willing or at all willing to restart treatment

*OP* osteoporosis

## Discussion

This study was designed to assess patients’ reasons for non-treatment of osteoporosis among patients who were never treated and reasons for discontinuation of osteoporosis therapy among those who had been treated previously. Currently and previously treated patients were older, more frequently had drug coverage through insurance, and reported better general health and fewer days of poor physical or mental health during the past 30 days than never-treated patients. These results are consistent with a US retrospective analysis that found that older age, better physical functioning, and prescription insurance coverage were among the patient characteristics associated with receiving osteoporosis therapy [13].

Among patients never treated for osteoporosis in the current study, the highest ranking reasons for non-treatment were the use of alternative treatments such as over-the-counter vitamins/supplements and the fear of side effects. The latter result agrees with the findings of Yu et al., who found that among US osteoporotic women aged 55 and older, the primary reason for not initiating osteoporosis therapy was concern over side effects (77.3 % of survey respondents) [4]. Similarly, in a survey that analyzed factors associated with the decision of US women to initiate osteoporosis therapy, respondents who did not initiate prescription medication for osteoporosis were more likely to worry about the side effects of taking osteoporosis medication and more likely to agree with the statement, “I can take care of my osteoporosis without medication” [14]. In addition, respondents who initiated osteoporosis prescription medication believed more strongly in the effectiveness of medication, while non-initiators reported having more distrust of medications [14]. In our analysis, fear of side effects was the most frequently first-ranked reason for non-treatment (see Fig. 1), and this fear may have acted as a driver for women to try alternative therapies, thus resulting in our finding that over half of the women included in this study preferred taking over-the-counter vitamins and supplements for osteoporosis.

The results of our survey show that the cost of medication is a barrier to initiating osteoporosis therapy among 24.1 % of the surveyed population. These results are consistent with those of Yu et al. Among the surveyed women, 34.1 % cited the cost of medication as their reason for not initiating treatment [4]. In addition, in a study of osteoporotic US women aged 40 and older, out-of-pocket spending on prescriptions as a proportion of income was found to be a significant predictor of receiving a prescription therapy for osteoporosis or osteopenia (*P* = 0.01) [13].

In the current survey, the most frequent reasons for discontinuation among previously treated patients included the direction of the physician, concerns about long-term safety, and the experience of side effects. The fact that some physicians are directing their patients to discontinue treatment is consistent with national guidelines that allow for a drug holiday [9], or it may be that physicians incorporate flexibility into the regimens of patients experiencing medication-related adverse effects. Experiencing side effects is a commonly cited reason for discontinuing osteoporosis therapy, as shown by health care and claims database analyses [6, 7] and telephone and questionnaire-based surveys [8, 15]. Regarding long-term safety profiles, patients taking bisphosphonates may be aware of reports of infrequent occurrences of renal toxicity, atypical subtrochanteric and femoral shaft fractures, osteonecrosis of the jaw, and, with intravenous formulations, influenza-like symptoms [16, 17]. An additional reason for discontinuation reported in this study was the cost of medication. This result is consistent with the findings of an Italian survey of postmenopausal women in which roughly 10 % of participants reported cost as a reason for discontinuation [18].

In the current study, 65.2 % of patients (195 of the 299 who answered this survey question) were somewhat to extremely willing to restart therapy. This value is higher than the rates of reinitiation reported in other observational studies [5, 6, 10, 11], perhaps because there is a difference between willingness to restart therapy and actually filling a prescription. Our results showed that patients not willing to restart osteoporosis treatment were more likely to have discontinued because of side effects and concerns about safety, compared to patients who were willing to restart therapy. These findings provide a link between reasons for discontinuation and predictors of reinitiation and offer insight into the known correlates of reinitiation of therapy. For example, women with side effects and women concerned about the long-term safety of their treatment will likely discontinue sooner, and early discontinuation is predictive of a lack of reinitiation of therapy [5, 10].

These findings have implications for insurance coverage of osteoporosis therapy. In order to manage pharmacy costs, managed care organizations may implement step-therapy restrictions in which patients are required to try generic, payer-preferred treatments before being reimbursed for a more expensive or branded medication. In many cases, this may mean that patients need to restart their previous medication in order to get a different prescription. Our study shows that treatment side effects may affect patient’s willingness to participate in the step-therapy process.

The primary limitation of this study is its potential lack of generalizability, because the survey population was derived from a consumer panel and may differ from patient populations derived from clinics or claims databases. In addition, the survey was conducted online which may also impact the generalizability. The cross-sectional survey design is limited by the accuracy of patient recall and reporting bias. For example, the diagnosis of osteoporosis in this study was based on self-report and not confirmed by medical records or physician contact and thus was subject to error. The lack of precision regarding the diagnosis of osteoporosis may explain why one quarter of patients included in our survey believed that osteoporosis was not serious enough to require a prescription medication. In addition, when participants were asked if they had ever had a BMD test or DEXA scan, recall bias and lack of knowledge of terminology could have impacted their response to this question. Also, in classifying patients as never treated, there was no distinction between patients who never received a prescription and those who received one but did not fill it. In this survey, no distinctions were made between types of osteoporosis treatments, so we could not determine whether patients taking oral versus injectable therapies or bisphosphonates versus non-bisphosphonates would have had substantially different results. In addition, we did not collect data on the time from osteoporosis diagnosis to medication initiation. This data may have helped determine whether patients that were never treated were simply not yet treated. Finally, the effect of confounders on the willingness to restart therapy was not assessed in this study.

## Conclusion

The results of this study show that barriers to osteoporosis therapy include a preference for alternative, non-prescription treatments and a fear of possible side effects. Furthermore, side effects are one of the most common reasons for discontinuing osteoporosis medications, and they appear to be associated with a lack of willingness to restart treatment.

## Electronic supplementary material

Below is the link to the electronic supplementary material.

ESM 1 (DOCX 48 kb)
