# Supplementary material for: Patient-reported barriers to osteoporosis therapy
Source: Arch Osteoporos. 2016 Apr 29;11(1):19. doi: 10.1007/s11657-016-0272-5 (PMC4851700; doi:10.1007/s11657-016-0272-5)
Supplement: Supplementary file 1 — (DOCX 48 kb) [file 11657_2016_272_MOESM1_ESM.docx]

**Section 1: Screener**

**S1.** Our first few questions will help us determine which questions to ask you.

In which country or region do you currently reside? [TERMINATE IF NOT US]

**S2.** In what state or territory do you currently reside?

**S3.** Are you…? (Male [TERMINATE]/Female)

S4. In what year were you born? Please enter your response as a four-digit number (for example, 1977).

[ TERMINATE IF UNDER 18]

**S7**. Are you post-menopausal (i.e., have you stopped having menstrual periods)? (Yes/No [TERMINATE])

**S8a.** Have you been told by a doctor or other healthcare provider that you have osteoporosis (T-score of less than -2.5)? (Yes/No)

**S8b.** Have you been told by a doctor or other healthcare provider that you have osteopenia or less severe thinning of the bones (T-score between -1.0 and -2.5) (Yes/No [TERMINATE])

**S10.** At what age were you first diagnosed with osteoporosis?

**S12.** We would like to know more about your experience with osteoporosis medications, so please feel free to gather your medication to consult the medication label.

Using the following chart, please select all of the medications that you currently use, have used in the

past, or never used to treat osteoporosis. *Please select only one option per medication.* (Oral osteoporosis treatments (such as Fosamax, Boniva, or Actonel), injectable osteoporosis treatments (such as Forteo or Prolia), Or other prescription osteoporosis medications (such as Evista).

**S13**. Has a healthcare provider ever given you a prescription for osteoporosis medication? (Yes/No)

**BASE: GIVEN OSTEOPOROSIS PRESCRIPTION**

**S14.** Did you fill/purchase the prescription that was given to you? (Yes, No, Not sure)

**BASE: TAKEN AN OSTEO MEDICATION IN THE PAST**

**S16.** Have you ever stopped taking a prescription medication for your osteoporosis without your doctor telling you to do so? (Please include any medications that you stopped taking without your doctor telling you to do so, even if you subsequently restarted the same or a similar therapy at some point in the past year). (Yes/No)

**SECTION 2: REASONS FOR CURRENT USE/ NONFULFILLMENT/ NON-PERSISTING**

**BASE: NON-TREATERS**

**Q1**. Why did you never start treatment for osteoporosis? *Please check all that apply.* [RANDOMIZE] [MULTIPLE RESPONSE]

1. I did not believe that my osteoporosis was serious/ life-threatening enough to take a prescription medication
2. Fear of side effects
3. Inconvenient/complex dosing regimen
4. Cost of medication
5. I was concerned about taking an osteoporosis prescription medication for the rest of my life
6. I was concerned about interactions with other medications I’m taking
7. I did not think the prescription medication would work for me/be effective for my osteoporosis
8. I decided to try life-style changes (diet and exercise) instead of taking osteoporosis prescription medications
9. I did not understand why my health care provider prescribed the osteoporosis medication for me
10. I decided to take over-the-counter vitamins and supplements for my osteoporosis instead of prescription medications
11. I felt like I already take too many medications and did not want to take any more
12. I thought I was too old to benefit from osteoporosis prescription medications
13. I heard negative news reports about osteoporosis medications
14. I knew family or friends who had bad experiences with osteoporosis prescription medications
15. Other, please specify

**BASE: NON-TREATERS**

**Q2**. What are your primary reasons for never starting prescription treatment for osteoporosis?

1. I did not believe that my osteoporosis was serious/ life-threatening enough to take a prescription medication
2. Fear of side effects
3. Inconvenient/complex dosing regimen
4. Cost of medication
5. I was concerned about taking an osteoporosis prescription medication for the rest of my life
6. I was concerned about interactions with other medications I’m taking
7. I did not think the prescription medication would work for me/be effective for my osteoporosis
8. I decided to try life-style changes (diet and exercise) instead of taking osteoporosis prescription medications
9. I did not understand why my health care provider prescribed the osteoporosis medication for me
10. I decided to take over-the-counter vitamins and supplements for my osteoporosis instead of prescription medications
11. I felt like I already take too many medications and did not want to take any more
12. I thought I was too old to benefit from osteoporosis prescription medications
13. I heard negative news reports about osteoporosis medications
14. I knew family or friends who had bad experiences with osteoporosis prescription medications
15. Other, please specify

**BASE: NON-TREATERS**

**Q3**. Why didn’t you fill/purchase the osteoporosis prescription medication? *Please check all that apply.* [RANDOMIZE] [MULTIPLE RESPONSE]

1. It wasn’t covered by my insurance
2. I did not believe that my osteoporosis was serious/ life threatening enough to take a prescription medication
3. Fear of side effects
4. Inconvenient/complex dosing regimen
5. Cost of medication
6. I was concerned about the taking an osteoporosis prescription medication for the rest of my life
7. I was concerned about interactions with other medications I’m taking
8. I did not think the prescription medication would work for me/be effective for my osteoporosis
9. I did not understand the purpose of the osteoporosis prescription medication
10. I decided to take over-the-counter vitamins and supplements for my osteoporosis instead of osteoporosis prescription medications
11. I decided to try life-style changes (diet and exercise) instead of taking osteoporosis prescription medications
12. I felt like I already take too many medications and did not want to take any more
13. I thought I was too old to benefit from osteoporosis prescription medications
14. I heard negative news reports about osteoporosis medications
15. I knew family or friends who has bad experiences with osteoporosis prescription medications
16. Other, please specify

**BASE: NON-TREATERS**

**Q4**. What are your primary reasons for not filling/purchasing the osteoporosis prescription medication?

1. It wasn’t covered by my insurance
2. I did not believe that my osteoporosis was serious/ life threatening enough to take a prescription medication
3. Fear of side effects
4. Inconvenient/complex dosing regimen
5. Cost of medication
6. I was concerned about taking an osteoporosis prescription medication for the rest of my life
7. I was concerned about interactions with other medications I’m taking
8. I did not think the prescription medication would work for me/be effective for my osteoporosis
9. I did not understand the purpose of the osteoporosis prescription medication
10. I decided to take over-the-counter vitamins and supplements for my osteoporosis instead of osteoporosis prescription medications
11. I decided to try life-style changes (diet and exercise) instead of taking osteoporosis prescription medications
12. I felt like I already take too many medications and did not want to take any more
13. I thought I was too old to benefit from osteoporosis prescription medications
14. I heard negative news reports about osteoporosis medications
15. I knew family or friends who has bad experiences with osteoporosis prescription medications
16. Other, please specify

**BASE: DID NOT FILL NEW OSTEOPOROSIS PRESCRIPTION OR STOPPED TAKING MEDICATION WITHOUT DOCTOR APPROVAL**

**Q5.** Earlier you mentioned you [“did not fill/purchase”; “stopped taking”] your osteoporosis medication. Did you tell the health care provider who prescribed you the osteoporosis prescription medication that you [“did not fill/purchase”; “stopped taking”] the medication? (Yes/No)

**BASE: ALL QUALIFIED RESPONDENTS**

**Q6.** Did the healthcare provider who prescribed your osteoporosis medication ask you about your medications at your last visit (e.g., if you filled the prescription, if you were still taking it, if you experienced side effects)? (Yes, no, not sure, I have not seen my healthcare provider since I was given my prescription)

**BASE: PREVIOUS TREATERS**

**Q7.** Why did you stop taking the osteoporosis prescription medication? *Please check all that apply.*

[RANDOMIZE] [MULTIPLE RESPONSES]

1. It wasn’t covered by my insurance
2. I did not believe that my osteoporosis was serious enough to take a prescription medication
3. I experienced side effects
4. Fear of side effects
5. Inconvenient/complex dosing regimen
6. Problems remembering to take it
7. Cost of medication
8. I was concerned about the long-term safety of osteoporosis prescription medications
9. I was concerned about interactions with other medications I’m taking
10. I did not think the prescription medication would work for me/was effective in treating my
11. I did not understand the purpose of the osteoporosis prescription medication
12. I did not believe that my osteoporosis was life threatening
13. I did not think that I needed the osteoporosis prescription medication
14. I decided to try life-style changes (diet and exercise) instead of taking osteoporosis prescription medications
15. I did not understand why my health care provider prescribed the osteoporosis prescription medication for me
16. I decided to take over-the-counter vitamins and supplements for my osteoporosis instead of prescription medications
17. I felt like I already take too many medications and did not want to take any more
18. My health care provider told me to stop taking the osteoporosis prescription medication
19. I heard negative news reports about osteoporosis medications
20. I knew family or friends who has bad experiences with osteoporosis prescription medications
21. Other, please specify

**BASE: PREVIOUS TREATERS**

**Q8**. What are your primary reasons for no longer taking the osteoporosis prescription medication?

1. It wasn’t covered by my insurance
2. I did not believe that my osteoporosis was serious enough to take a prescription medication
3. I experienced side effects
4. Fear of side effects
5. Inconvenient/complex dosing regimen
6. Problems remembering to take it
7. Cost of medication
8. I was concerned about the long-term safety of osteoporosis prescription medications
9. I was concerned about interactions with other medications I’m taking
10. I did not think the prescription medication would work for me/was effective in treating my
11. I did not understand the purpose of the osteoporosis prescription medication
12. I did not believe that my osteoporosis was life threatening
13. I did not think that I needed the osteoporosis prescription medication
14. I decided to try life-style changes (diet and exercise) instead of taking osteoporosis prescription medications
15. I did not understand why my health care provider prescribed the osteoporosis prescription medication for me
16. I decided to take over-the-counter vitamins and supplements for my osteoporosis instead of prescription medications
17. I felt like I already take too many medications and did not want to take any more
18. My health care provider told me to stop taking the osteoporosis prescription medication
19. I heard negative news reports about osteoporosis medications
20. I knew family or friends who has bad experiences with osteoporosis prescription medications
21. Other, please specify

**BASE: CURRENTLY TREATING**

**Q9a.** Using the scale below (Disagree completely, Disagree mostly, Disagree somewhat, Agree somewhat, Agree mostly, Agree completely), please indicate how much you agree or disagree with the following statements about your current osteoporosis medication. (I am convinced of the importance of my osteoporosis prescription medication, I worry that my osteoporosis medication will do more harm than good to me, I feel financially burdened by my out-of-pocket expenses for my osteoporosis medication)

**BASE: CURRENTLY TREATING**

**Q9b.** Using the scale below (strongly disagree, somewhat disagree, neither agree nor disagree, somewhat agree, strongly agree), please indicate how much you agree or disagree with the following statements about your current osteoporosis medication. (My osteoporosis medication is effective in managing my osteoporosis, My osteoporosis medication regimen disrupts my life, The potential side effects of osteoporosis prescription medication worry me, My osteoporosis medication allows me to maintain an active lifestyle, My osteoporosis medication is a convenient way to treat my osteoporosis)

**BASE: CURRENTLY TREATING**

**Q10.** In the past 30 days, thinking of the medications prescribed to you by your doctor(s) for your osteoporosis, please answer the following questions. (Never, Rarely, Sometimes, Often, Always, Not applicable)

1. Do you sometimes forget to take your osteoporosis medication?
2. Do you miss taking your medications for reasons other than forgetting?
3. Have you ever cut back or stopped taking your osteoporosis medication without telling your doctor?
4. Do you ever travel or leave home and forget to bring along your osteoporosis medication?
5. If you think your osteoporosis medication is not working, do you sometimes stop taking your medication?
6. Do you ever feel hassled about sticking to your osteoporosis treatment plan?
7. How often do you have difficulty remembering to take all your medications?

**BASE: CURRENTLY TREATING**

**Q10A.** In the past 30 days, thinking of the medications prescribed to you by your doctor(s) for your osteoporosis, did you take your osteoporosis medication the last time you were supposed to take it? (Yes/No)

**BASE: CURRENTLY TREATING**

**Q11.** In the past two years did you take another osteoporosis medication prior to the one you are currently taking? (Yes/No)

**BASE: SWITCHED MEDICATIONS**

**Q12.** What was your primary reason for switching to the osteoporosis medication you are currently taking? [RANDOMIZE]

1. The cost of the medication
2. The medication was difficult or inconvenient to get
3. The medication was inconvenient to take as directed (e.g. staying upright and not eating or drinking for 30 minutes after taking the medication)
4. I had difficulty taking the medication at the specific times I was supposed to
5. I had too many pills/medications to take
6. The medication was not effective in controlling my osteoporosis
7. I experienced side effects (e.g., too many or too severe, made me feel bad)
8. I learned/heard of potential side effects or other negative things about the medication
9. Other, please specify

98 None of these

**Section 3: Osteoporosis Care/HCP Relationships**

**Base: All Qualified Respondents**

**Q13.** What is the specialty of the physician who initially told you that you have osteoporosis? (Primary care physician, Gynecologist, Rheumatologist, Endocrinologist, Orthopedist, Other physician)

**Base: All Qualified Respondents**

**Q13A.** What is the specialty of the physician who currently treats your osteoporosis? (Primary care physician, Gynecologist, Rheumatologist, Endocrinologist, Orthopedist, Other physician, I am not currently being treated by a physician)

**Base: Currently being Treated by a Physician for Osteoporosis**

**Q13B.** Have you seen this physician in the past 12 months? (Yes/No)

**BASE: Seen Physician in Past 12 Months**

**Q15.** We would like to learn about the type of help you receive from or the things you talk about with your physician during visits for your osteoporosis.

Over the past 12 months, when you received care from your healthcare provider, how often did you do the following things? (Never, A little of the time, Some of the time, Most of the time, Always) [RANDOMIZE]

1. We discussed how my osteoporosis affects my life
2. We discussed my ability to afford my osteoporosis medications
3. We talked about whether I had any problems with my osteoporosis medications or any side effects
4. We discussed the importance of adherence to my osteoporosis treatment regimen
5. We discussed my system for adhering to my osteoporosis treatment regimen (e.g., calendars, reminders from family/caregivers)

**BASE: Seen Physician in Past 12 Months**

**Q16.** Over the past 12 months, who usually initiated the discussion about the following? (I did, my physician did, we did not discuss, not sure) [RANDOMIZE]

1. Test results
2. Your satisfaction with your osteoporosis treatment regimen
3. Side effects of your osteoporosis medications
4. Other health conditions
5. New treatment options for osteoporosis
6. The importance of adherence to your osteoporosis treatment regimen

**BASE: Seen Physician in Past 12 Months**

**Q17.** Please indicate how often the following statements are true regarding your visits with the physician who treats your osteoporosis over the past 12 months. (Never, Rarely, Sometimes, Often, Always) [RANDOMIZE]

1. I felt understood by my physician
2. I was able to be open with my physician at our appointments
3. My physician conveyed confidence in my ability to make lifestyle changes to take my osteoporosis treatment regimen as prescribed
4. My physician encouraged me to ask questions
5. I have a lot of trust in my physician
6. My physician cares about me

**Base: Seen Physician in Past 12 Months**

**Q19.** Please rate how well your doctor performed on the following actions related to your osteoporosis treatment over the past 12 months. (Poor, Fair, Good, Very good, Excellent) [RANDOMIZE]

1. Showing concern about my osteoporosis
2. Offering different treatment options for my osteoporosis
3. Recommending prescription medication treatment options for my osteoporosis
4. Telling me everything I need to know about osteoporosis
5. Discussing pros and cons of different treatment options with me
6. Helping me understand what to expect in the future with my osteoporosis

**Section 4: Attitudes and Beliefs about Osteoporosis**

**Base: All Qualified Respondents**

**Q20.** Please rate your level of agreement when it comes to the following statements about treating your osteoporosis. (Disagree completely, Disagree mostly, Disagree somewhat, Neither agree nor disagree, Agree somewhat, Agree mostly, Agree completely) [RANDOMIZE]

1. I believe lifestyle changes (such as diet and exercise) are enough to help me deal with my osteoporosis
2. I am likely to experience negative side effects from prescription drugs for my osteoporosis
3. The benefits of taking osteoporosis medications outweigh the risks
4. I don’t want to take medications for my osteoporosis unless I absolutely have to
5. I worry about the negative side effects of prescription medications for osteoporosis
6. Prescription medications for osteoporosis will help to prevent fractures
7. Taking prescription medications for osteoporosis can help me maintain my current lifestyle
8. I believe that vitamin, mineral, and herbal supplements can help my osteoporosis more than prescription medications can
9. I prefer to treat my osteoporosis with natural remedies rather than prescription medications
10. I faithfully take vitamins and supplements for my osteoporosis

**Base: All Qualified Respondents**

**Q21.** Please indicate which of the following statements are true related to your physician’s recommendations for treating your osteoporosis. *Please check all that apply.* [RANDOMIZE] [MULTIPLE RESPONSE]

1. My doctor recommended that I increase my intake of calcium to help my osteoporosis
2. My doctor recommended that I increase my intake of vitamin D to help my osteoporosis
3. My doctor recommended that I improve my nutrition to help my osteoporosis
4. My doctor recommended that I exercise more to help my osteoporosis
5. None of these

**Base: All Qualified Respondents**

**Q22.** In your opinion, how serious as a health condition is osteoporosis compared to each of the following other conditions? (Mark only one box for each statement.) (Osteoporosis is less serious than, Osteoporosis is about as serious as, Osteoporosis is more serious than) [RANDOMIZE]

1. Alzheimer's disease
2. High blood pressure
3. Breast cancer
4. Coronary heart disease
5. High cholesterol
6. Diabetes
7. Stroke
8. Depression
9. Heart attack
10. GERD (reflux disease)
11. Asthma
12. Osteoarthritis

**Base: All Qualified Respondents**

**Q23.** Please rate your level of agreement when it comes to the following statements about osteoporosis. (Disagree completely, Disagree mostly, Disagree somewhat, Neither agree nor disagree, Agree somewhat, Agree mostly, Agree completely) [RANDOMIZE]

1. I take my diagnosis of osteoporosis very seriously
2. Osteoporosis is a threat to my health and well-being
3. Osteoporosis is not a major medical concern for me
4. Osteoporosis is not something I need to treat right now
5. Osteoporosis is not as urgent of a health concern as other conditions I have
6. I have heard conflicting information about the treatment of osteoporosis
7. As time goes on, my osteoporosis will get worse
8. I experience symptoms from my osteoporosis
9. I think about my osteoporosis every day
10. Osteoporosis is hindering my lifestyle

**Base: All Qualified Respondents**

**Q24.** Please indicate whether the following statements about osteoporosis are true or false. If you are unsure, please provide your best guess. (True/False) [RANDOMIZE]

1. Only older women get osteoporosis
2. Osteoporosis makes bones weak
3. Osteoporosis causes bones to break more easily
4. People with osteoporosis do not have any symptoms unless they break a bone
5. There is little that can be done to prevent osteoporosis after menopause.
6. Someone can have osteoporosis for years without knowing it until a bone is broken
7. If someone has osteoporosis, something as simple as coughing or lifting a bag of groceries can cause a bone to break
8. A good diet can reduce the chance of developing osteoporosis.
9. Physical exercise can reduce the chance of developing osteoporosis.
10. There is little that can be done to prevent osteoporosis
11. Loss of height naturally occurs with age and is not a sign of osteoporosis
12. Cigarette smoking can contribute to osteoporosis
13. Alcohol use has little effect on osteoporosis

**Base: All Qualified Respondents**

**Q25.** Please rate your level of agreement regarding the potential for fractures due to osteoporosis. (Disagree completely, Disagree mostly, Disagree somewhat, Neither agree nor disagree, Agree somewhat, Agree mostly, Agree completely) [RANDOMIZE]

1. Having osteoporosis increases my chance of breaking my hip or wrist if I fall
2. If I were to break my hip, I might never fully recover
3. The consequences of a fracture are severe
4. I am concerned about having a fracture

**BASE: ALL QUALIFIED RESPONDENTS**

**Q26.** Please indicate which of the following factors is most important and which is least important when you are choosing n prescription osteoporosis treatment. Please review each set of 4 factors and choose the one that is most important and the one that is the least important.

1. Has few to no drug interactions with other medications I’m taking
2. Is affordable every month (I am able pay for my prescription)
3. Fits well with my lifestyle
4. Does not cause severe daily side effects
5. Does not cause long-term side effects
6. Has a simple/convenient dosing regimen
7. I have friends or family members who have taken it before
8. My doctor recommended it
9. It has no/few administration restrictions (timing, food, activity, etc.)
10. Is effective in managing my osteoporosis

**Base: Previously Took Oral Prescription Medication**

**Q27.** In order to contain costs, insurance companies may employ rules about which medications can be prescribed and in what order. For instance, sometimes insurance companies require that patients try a less expensive or generic medication first, and then progress to more expensive or branded medications.

You mentioned that you previously took an oral prescription medication for your osteoporosis. How willing would you be to try taking the oral drug you previously used in the past for a short period of time in order to be eligible for insurance coverage of a more expensive medication for treatment of your osteoporosis? (Not at all willing, Somewhat willing, Willing, Very willing)

Section 5: Demographics

**BASE: ALL QUALIFIED RESPONDENTS**

**D1.** In the **past 12 months**, have you fallen? (Yes/No)

**BASE: FELL IN PAST 12 MONTHS**

**D2.** Did any fall **in the past 12 months** result in a broken or fractured bone? (Yes/No)

**BASE: ALL QUALIFIED RESPONDENTS OLDER THAN 45**

**D3.** Have you **ever** broken or fractured any bone ***after the age of 45***? (Yes/No)

**BASE: HAS BROKEN/FRACTURED A BONE AFTER AGE 45**

**D4.** Please tell us which bone(s) you have *broken* ***after the age of 45.*** (Hip, Spine (backbone or vertebrae, wrist, rib, or other bone (e.g., ankle, pelvis, forearm, upper arm))

**BASE: ALL QUALIFIED RESPONDENTS**

**D5.** Have you **ever** had a bone mineral density (BMD) test (also called a DEXA scan)? (Yes/No)

**BASE: HAS HAD A BMD TEST**

**D6.** Did your doctor discuss the results of your last BMD test with you? (Yes/No)

**BASE: ALL QUALIFIED RESPONDENTS**

**D7.** Please indicate if you currently have any of the following conditions. For conditions which you currently have, please indicate if you are currently taking prescription medication for that condition. (Heart Disease, COPD, Connective Tissue Disease, Peptic Ulcer Disease, Diabetes Mellitus, Moderate to Severe Chronic Kidney Disease, Cancer, Liver Disease, AIDS, High Cholesterol, High Blood Pressure, Depression, Acid Reflux, Migraines [ALPHEBETIZE])

BASE: ALL QUALIFIED RESPONDENTS

D8. Would you say that in general your health is excellent, very good, good, fair or poor?

**BASE: ALL QUALIFIED RESPONDENTS**

**D9.** Now thinking about your physical health overall, which includes physical illness and injury, how many days during the past 30 days was your physical health not good? [INSERT NUMERIC TEXT BOX] [RANGE: 0-30]

**BASE: ALL QUALIFIED RESPONDENTS**

**D10.** Now thinking about your mental health overall, which includes stress, depression, and problems with emotions, how many days during the past 30 days was your mental health not good?[INSERT NUMBERIC TEXT BOX] [RANGE: 0-30]

**BASE: ALL QUALIFIED RESPONDENTS**

**D11.** During the past 30 days, approximately how many days did poor physical or mental health keep you from doing your usual activities, such as self-care, work, or recreation? [INSERT NUMERIC TEXT BOX] [RANGE: 0-30]

BASE: ALL RESPONDENTS AGE 18+

D12. Are you of Spanish or Hispanic origin, such as Latin American, Mexican, Puerto Rican, or Cuban? (Yes, of Hispanic origin, No, not of Hispanic origin, or Decline to answer)

**BASE: ALL RESPONDENTS AGE 18+**

**D13.** Do you consider yourself…? (White, Black, Asian or Pacific Islander, Native American or Alaskan Native, African American, Mixed Race, Some other race, or Decline to answer)

BASE: OTHER RACE

**D14.** What other race do you consider yourself? [NON-MANDATORY TEXT BOX]

**BASE: MIXED RACIAL BACKGROUND**

**D15.** You indicated that you consider yourself of a mixed racial background. With which of the following racial groups do you most closely identify? *Please select all that apply.* (White, Black, Asian or Pacific Islander, Native American or Alaskan Native, African American, Mixed Race, Some other race, or Decline to answer [MULTIPLE RESPONSE])

**BASE: ALL RESPONDENTS 18+**

**D16.** Which of the following best describes your current work situation? (Work full time, Work part time, Not working, but looking for work, Not working and not looking for work, Unable to work, Retired, Student, or Stay-at-home spouse or partner)

**BASE: ALL RESPONDENTS 18+**

**D17.** What is the highest grade of school you have completed or the highest degree you have received? (8^th^ grade or less, Some high school, Completed high school, Some college/Associate Degree, Completed college, Some graduate school, Completed graduate school, or Other)

BASE: all respondents 18+

**D18.** Which of the following income categories best describes your total [INSERT LAST YEAR] household income before taxes? (Less than $15,000, $15,000 to $24,999, $25,000 to $34,999, $35,000 to $49,999, $50,000 to $74,999, $75,000 to $99,999, $100,000 to $124,999, $125,000 to $149,999, $150,000 to $199,999, $200,000 to $249,999, $250,000 or more, or Decline to answer)

BASE: all respondents 18+

**D19.** What type(s) of health insurance coverage/plan do you currently have? Please select all that apply. (Private insurance (PPO/ HMO/Managed Care), Medicare, Medicaid, Veteran’s Insurance, Other, I currently do not have health insurance/I pay cash in full for my health care, or Not sure)

BASE: all respondents 18+

**D20.** Do you currently have prescription drug coverage? (Yes, through my health insurance plan, Yes, through a patient assistance or co-pay card from a pharmaceutical company that provides discounts on medications, No, or Not sure)

**SECTION 1300: PROPENSITY QUESTIONS (All US respondents, aged ≥18)**

**Q1305.** On how many separate occasions would you say you watched news programs on TV during the past 30 days? [RANGE:  0-120].

**Q1310.** On how many different occasions did you do vigorous physical exercise during the past 30 days? [RANGE:  0-120]

**Q1315.** Do you think that community service should be a requirement in schools? (Yes/No)

**Q1320.** Have you chosen not to purchase a product or service or told someone not to purchase a product or service made by a particular company because the company’s actions or policies conflicted with your values or beliefs? (Yes, in the past year, Yes, more than 1 year ago, but within the past 2 years. Yes, more than 2 years ago, but within the past 3 years, Yes, more than 3 years ago, or No, I have never done this)

**Q1325.** Most companies today want to know about the individual interests and lifestyle of their customers so they can tailor their information services and products to each customer's personal preferences.  In general, do you see such personalization as a good thing? (Yes/No)
